# Using Social Media to Detect Outdoor Air Pollution and Monitor Air Quality Index (AQI): A Geo-Targeted Spatiotemporal Analysis Framework with Sina Weibo (Chinese Twitter)

**DOI:** 10.1371/journal.pone.0141185

**Published:** 2015-10-27

**Authors:** Wei Jiang, Yandong Wang, Ming-Hsiang Tsou, Xiaokang Fu

**Affiliations:** 1 State Key Laboratory of Information Engineer in Surveying, Mapping and Remote Sensing, Wuhan University, Wuhan, Hubei, China; 2 Department of Geography, San Diego State University, San Diego, California, United States of America; Northwestern University, UNITED STATES

## Abstract

Outdoor air pollution is a serious problem in many developing countries today. This study focuses on monitoring the dynamic changes of air quality effectively in large cities by analyzing the spatiotemporal trends in geo-targeted social media messages with comprehensive big data filtering procedures. We introduce a new social media analytic framework to (1) investigate the relationship between air pollution topics posted in Sina Weibo (Chinese Twitter) and the daily Air Quality Index (AQI) published by China’s Ministry of Environmental Protection; and (2) monitor the dynamics of air quality index by using social media messages. Correlation analysis was used to compare the connections between discussion trends in social media messages and the temporal changes in the AQI during 2012. We categorized relevant messages into three types, retweets, mobile app messages, and original individual messages finding that original individual messages had the highest correlation to the Air Quality Index. Based on this correlation analysis, individual messages were used to monitor the AQI in 2013. Our study indicates that the filtered social media messages are strongly correlated to the AQI and can be used to monitor the air quality dynamics to some extent.

## Introduction

In recent years, social media, such as Twitter, Facebook, and Sina Weibo (a popular Chinese version of social media synonymous to Twitter), has become a major communication channel in our society [1, 2]. Subsequently, these social media messages may help us monitor and detect dynamic changes in both cyberspace activities and our real-world environment [3]. Users of social media can be considered as “social sensors” while content from social media platforms can provide real-time updates of social activities and collective human behaviors [4].

Many researchers have applied social media to study various real world events, such as flu outbreaks [5–8], elections [9, 10], and earthquakes [11, 12]. For example, the frequency of flu and influenza keywords in Twitter messages has been compared with weekly hospital records of influenza-like illness (ILI) in different cities [5]. By filtering out invalid tweets, such as those related to flu shots and swine flu, this research demonstrated that occurrences of flu outbreaks in different cities strongly corresponds to trends in social media messages [5]. Social media analytics are regarded as an effective way to predict the results of elections [9, 10]. In the 2012 U.S. presidential election, the final results were predicted by comparing the trends in social media messages about two candidates [9]. In addition to disease outbreak monitoring and predicting election results, social media has been used to study natural disasters. In Japan, researchers found that a sudden increase in microblogging activities could be used to detect earthquake tremors in real-time [11, 12]. These studies indicate that social media messages in the cyberspace can be used to explore the issue in the real world.

Outdoor air pollution has become a more and more serious issue over recent years, especially in developing countries experiencing rapid population growth [13–15]. Previous studies within the fields of biology and environmental sciences have explored the impact of outdoor air pollution on various aspects of people’s lives [16–18]. A 2012 report published by the World Health Organization estimated that outdoor air pollution caused 3.7 million premature deaths all over the world [19]. To prevent exacerbation of air pollution, near real-time air quality monitoring has been established to measure pollution levels in many localities. Urban air quality is measured at air monitoring stations. Building air monitoring stations requires land, incurs costs and entails skilled technicians to maintain a station. As of May 2015, only 16700 monitoring stations have been built in 65 countries [20]. Many countries do not have any monitoring stations and even lack any means to monitor air quality [20]. Regardless of the presence of monitoring stations or not, in cities with serious air pollution, people use the social media to express their attitudes and feelings about it. Therefore, the social media could be used to monitor air quality dynamically. Wang et al. [21] have found that the air pollution related message volume in Sina Weibo is indicative of the annual particle pollution levels. They measured the Pearson correlation between message volume and annual particle pollution level in 74 cities. At a smaller time scale, Mei et al. [22] applied a Markov Random Field model, a machine learning method, to monitor the daily AQI by exploring the relationship between the terms in air pollution related messages and AQI. However, no studies have investigated the inter-correlations between real-space and cyberspace by examining variation in micro-blogging behaviors relative to changes in daily air quality. Thus, existing methods of monitoring AQI using micro-blogging data shows a high degree of error between real AQI and air quality as inferred from social media messages. In addition, users can be considered as “social sensors” [4] who post air pollution related messages on social media platforms; therefore, rich information about popular perceptions of air quality in the real world can be extracted from these messages.

In this paper, we introduce a new geo-targeted social media analytic method to (1) investigate the dynamic relationship between air pollution-related posts on Sina Weibo and daily AQI values; (2) apply Gradient Tree Boosting, a machine learning method, to monitor the dynamics of AQI using filtered social media messages. Simultaneously, we explore popular perceptions of air quality from the contents of messages. Our results expose the spatiotemporal relationships between social media messages and real-world environmental changes as well suggesting new ways to monitor air pollution using social media.

## Background

### Sina Weibo

Sina Weibo, established in 2009, is the largest microblogging service in China. Microblogging is one type of popular social media services that allows users to update brief content called “microblogs” in the form of short sentences, individual images, web page links, or video links [23]. Popular microblogging services include Twitter and Sina Weibo [24]. As of December 2012, the number of registered Sina Weibo users exceeded 500 Million, accounting for 57% of microblogging users in China [25, 26]. Active users can number 4.6 million daily with about 100 million messages posted every day [27]. Similar to Twitter’s messages called “tweets”, Sina Weibo users can only post their messages with a 140-Chinese-character limit. Each posted message in Sina Weibo is called a “weibo”. Sina Weibo functions are very similar to Twitter, such as retweets (RTs), mentioned (@), and hashtags (#). The major differences between Sina Weibo and Twitter are in the content available from user profiles. Sina Weibo collects more optional personal information, such as gender, user locations, birthdays, and blood type in user profiles. Sina Weibo provides powerful search engine application programming interfaces (APIs) for collecting and analyzing microblog messages. In this study, we collected Sina Weibo messages from APIs and used these messages to monitor outdoor air quality dynamically.

### Gradient Tree Boosting

Gradient Tree Boosting (GTB) is an algorithm for developing prediction models to solve classification and regression problems in data mining and machine learning: it iteratively builds a regression tree from residuals and outputs weighting sum of the regression trees [28]. We define the training samples including N pairs as ${\left\{y_{i},x_{i}\right\}}_{1}^{N}$. ***x*** is a set of explanatory variables (***x***_1_,***x***_2_,…,***x***_k_) and *y* is the target value. GTB constructs the following additive function *F*(***x***):

$$F(x)=\beta_{0}+\sum_{m=1}^{M}\beta_{m}h(x,a_{m})$$ (1)

where *β*_*m*_ and *β*_0_ are a weight for the *m*th regression tree *h*(***x***,***a***_*m*_) and an initial weight, respectively. ***a***_*m*_ is vector of parameters for *h*(***x***,***a***_*m*_). ***M*** is the number of iterations. To minimize the loss function *ψ*(*y*, *F*(***x***)), the *β*_*m*_ and ***a***_*m*_ are determined as follows:

$$(\beta_{m},a_{m})=\underset{\beta ,a}{\text{arg min}}\sum_{i=1}^{N}\psi (y_{i},F_{m-1}(x_{i})+\beta h(x_{i},a))$$ (2)

where *F*_m-1_(***x***) is a an additive function which is combined from the first regression tree to the (*m*-1)th regression tree. Because Eq (2) is difficult to compute, Friedman [29] proposed a two-step procedure; first, the ***a***_*m*_ for regression tree is decided as follows:

$$a_{m}=\underset{a}{\text{arg min}}\sum_{i=1}^{N}{\left({\tilde{y}}_{im}-h(x_{i},a)\right)}^{2}$$ (3)

where ${\tilde{y}}_{im}$ is the gradient and is decided by

$${\tilde{y}}_{im}=-{\left[\frac{\partial \psi (y_{i},F(x_{i}))}{\partial F(x_{i})}\right]}_{F(x)=F_{m-1}(x)}$$ (4)

Secondly, the optimal value of *β*_*m*_ is determined as:

$$\beta_{m}=\underset{\beta }{\text{arg min}}\sum_{i=1}^{N}\psi (y_{i},F_{m-1}(x_{i})+\beta h(x_{i},a_{m}))$$ (5)

when the *m*th regression tree using the ***a***_*m*_ has *L*_*m*_ leaf nodes, the regression is given by

$$h(x,{\left\{R_{lm}\right\}}_{l=1}^{L_{m}})=\sum_{l=1}^{L_{m}}{\bar{y}}_{lm}1(x\in R_{lm})$$ (6)

where 1(•) is a Boolean function that outputs 1 in case the argument of the function is true. At each iteration *m*, a regression tree partitions the ***x*** space into *L* disjoint regions ${\left\{R_{lm}\right\}}_{1}^{L}$ [30]. ${\bar{y}}_{lm}$is defined as the mean of training data that belongs to the *lm*th region. The additive function *F*_*m*_(***x***) can be updated as follows:

$$F_{m}(x)=F_{m-1}(x)+v\sum_{l=1}^{L_{m}}\beta_{m}{\bar{y}}_{lm}1(x\in R_{lm})$$ (7)

where *v* is the learning rate. Compared to other regression algorithms, the advantage of GTB is that the algorithm can be implemented quickly and avoids overfitting [31]. In this study, GTB was applied to monitor the AQI using filtered social media messages.

## Data Collection

**Fig 1:**
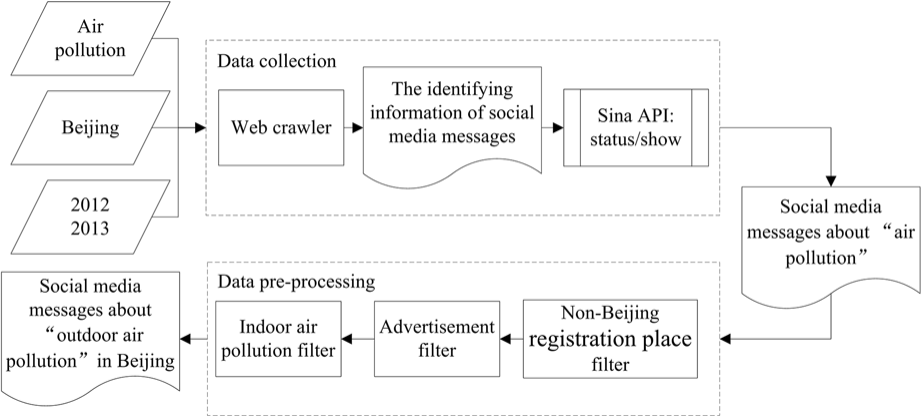
The framework for obtaining social media messages about “outdoor air pollution”. The framework containing two phases: data collection and data pre-processing.

Two types of data were used in this study. One type was the air quality index (AQI) data obtained from China’s Ministry of Environmental Protection. The AQI data were the daily pollution measurement announcements for Beijing. The other type of data was the social media messages about “outdoor air pollution”. To collect these messages, a framework containing both data collection and data pre-processing phases was designed, as shown in Fig 1. The details of these two phases are introduced as follows:

(1) In the data collection phase, we collected detailed information from social media messages about “air pollution” using Sina API and a web crawler that simulated an “advanced search”. Sina Weibo provides the “advanced search” interface that can be used to search the social media messages based on keywords, registration profile, and posting time. The “advanced search” interface can only show partial information from social media messages; this information cannot be downloaded. Because of these limitations of the “advanced search function”, we designed a detailed message collection method consisting of two steps. First, a web crawler was developed to simulate the “advanced search” interface and then store the identifying information from the social media messages. Second, detailed information in the social media messages was collected using the Sina API, “statuses/show”; that obtained social media messages based on the stored identifying information. In this study, we assigned “空气污染” (air pollution) as the keyword and “北京” (Beijing) as the place of registration for users to obtain the social media messages posted between January 1, 2012 and December 31, 2013. A total of 179,316 (Weibo) messages were obtained through this collection method. Each message contained several attributes, such as the time of the microblog posting, the microblog text, the place of registration, reposting information, and source.

(2) In the data pre-processing phase, we obtained social media messages about “outdoor air pollution” in Beijing by filtering out noise. The noise was messages related to ***non-Beijing registration places***, ***indoor air pollution*** and ***advertisements***. Although we collected the data based on the user registration places, the data still contained some messages whose registration places were not “北京” (Beijing). To obtain the data only within the city of Beijing, 40,364 messages with non-Beijing registration places were filtered out. By analyzing the texts of the remaining noise after filtering, we found that most of the microblogs about indoor air pollution had particular words, such as “室内” (indoor) and “甲醛” (formaldehyde), and advertisements (usually robot-generated messages) relevant to air pollution with particular symbols, such as “【】”. We removed 21,691 Weibo messages containing these particular words and symbols.

## Categorization and Content Analysis of Microblogs

To extract data content about the dynamics of air quality in the real world, we categorized the microblogs into three types: 1) retweet messages, 2) mobile app messages, and 3) original individual messages. For each type of microblog, the frequency of the vocabulary concerning air pollution that occurred in the microblog texts was calculated. These words in turn, were used to aggregate and analyze the contents of the collected Sina Weibo messages.

### (1) Retweet Messages

The term, retweet messages, refers to those microblogs reposting the contents of other microblogs [32, 33]. In Sina Weibo, if a microblog reposts other microblogs, there will be a reposting symbol included. We extracted the microblogs with reposting symbols as retweets. Most retweet messages were promoted by celebrity microblogs. To explore the contents of retweet messages, the top ten most frequently appearing terms on Sina Weibo were calculated. The names of two celebrities “薛蛮子” (Charles Xue) and “潘石屹” (Shiyi Pan) appeared among these terms. Together with celebrity names, the words “国家” (nation) and “政府” (government) also appeared, suggesting that celebrities appealing to the government to prevent the deterioration of air quality are supported by the many people retweeting their messages.

### (2) Mobile App Messages

Mobile app messages are microblogs posted by app users. Mobile apps (application software) are computer programs designed to run on smartphones, tablet computers, and other mobile devices [34]. Many people install air pollution apps on their mobile phones. These apps broadcast air pollution updates containing the AQI every few hours and allow users to post these updates on Sina Weibo directly. Updates containing very good or bad air quality information could prompt users to post these updates on Sina Weibo. The sources of these microblogs are termed air pollution apps. Based on the sources, we extracted mobile app messages. These messages are similar to the concept of “retweets”, but the original messages are generated by the air pollution apps automatically rather than by individuals. By analyzing the top ten most frequently occurring terms mentioned on mobile app messages, we found that many of the frequently occurring terms came from the contents of updates as announced by apps, such as “微风” (breeze), “毒害” (poison), “优” (excellent) and “颗粒物” (particular matter).

### (3) Original Individual Messages

Original individual messages are created by social media users expressing their own personal opinions. Individual users mentioned air pollution keywords in their individual messages in relation to air quality around their own immediate surroundings. We extracted the microblogs that were not retweets and not mobile app messages and termed them individual messages. These messages had very different contents than retweets and mobile app messages. The top ten most frequently occurring terms mentioned in individual messages were calculated. An analysis of frequently occurring terms showed that many individual messages had contents concerning issues related to air pollution. For example, “问题” (issue), “污染物” (pollutant), “环境” (environment) and “帝都” (capital) were descriptions of air pollution in Beijing. “口罩” (mask) referred to protective gear while “肺癌” (lung cancer) suggested popular awareness of the health risks associated with air pollution.

Table 1 illustrates the different types of messages relevant to air pollution topics. We found that original individual messages accounted for 15.83%, while mobile app messages represented 32.96% of all messages. The retweet messages constituted 16.60% of the total.

**Table 1:** Different types of microblogs collected in Beijing relevant to air pollution.

	Numbers	Percentages	Notes
**Mobile App Messages**	59,108	32.96%	Reposting from mobile apps
**Retweet Messages**	29,772	16.60%	Reposting from other individuals
**Original Individual Messages**	28,381	15.83%	Generated by individuals
**Noise**	62,055	34.61%	Non-Beijing registration places, indoor air pollution and advertisements
**Total**	179,316	100.00%	—

The number and percentage of each type of microblogs.

## Analysis of Different Kinds of Microblogs

In this section, we investigated how the dynamics of air quality in the year 2012 was revealed in cyberspace, as measured by different types of microblogs. In particular, we focused on a correlation analysis between the air quality in the real world and 2012 filtered social media messages. Because the air pollution could change obviously from month to month, the correlation was analyzed on the per-month base.

### Comparing original individual messages to AQI

We explored the relationship between the temporal trends in individual messages and the dynamic changes of the daily AQI by using the Pearson correlation coefficient. Two steps were required to investigate their relationship: (1) determining the Pearson correlation coefficients between the daily frequency of individual messages and the AQI, and (2) determining the Pearson correlation coefficient between the negative individual messages and the AQI.

**Fig 2:**
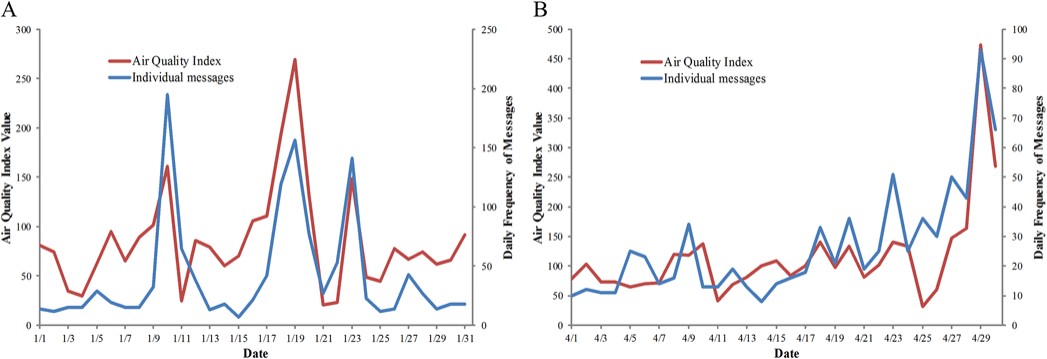
Examples of high correlations between AQI and individual messages. (A) The trends of AQI (red line) and individual messages (blue line) for January 2012 in Beijing. (B) The trends of AQI (red line) and individual messages (blue line) in April 2012 in Beijing.

Based on the correlation analysis between the daily frequency of individual messages about pollution and daily AQI for each month in the year 2012, we found that the dynamics of AQI were consistent with the pattern of individual messages for most months. The Pearson correlation coefficients between the AQI and the total microblogs (combining individual messages, retweets, and mobile app messages) and individual messages were denoted as r_t_ and r_i_. The r_t_ and the r_i_ for each month are shown in Table 2. Most values of r_i_ were higher than the r_t_. This demonstrates that, as compared to the total microblogs, the individual messages were more strongly correlated to the AQI. The r_i_ obtained for the entire year was 0.57 and significant at p < 0.01. This value was very close to the average value of r_i_ obtained for each month and could be considered as the comprehensive representation for 12 months. In the Table 2, the values of r_i_ for seven months were higher than 0.6, and significant at p < 0.01. In the months with the high values of r_i_, individual messages were strongly correlated to the AQI and were able to reflect the dynamic changes in the AQI. For example, values of r_i_ in January and April were 0.75 and 0.83; the trends for individual messages and AQI for these two months were consistent, Fig 2. Individual messages were expression of personal opinions about air pollution. Thus, as the AQI increased, the frequency of individual messages might also increase. In the months with the low values of r_i_, the individual messages were not strongly correlated to the AQI. In next section, we will focus on how to improve the correlation results.

**Table 2:** Correlation coefficient between AQI and individual messages.

	The Pearson correlation coefficient between AQI and the total microblogs	The Pearson correlation coefficient between AQI and the total individual messages	The largest AQI value	The range of AQI
**January**	0.70**	0.75**	269	21~269
**February**	0.29	0.65**	152	26~152
**March**	0.27	0.73**	159	30~159
**April**	0.23	0.83**	473	32~473
**May**	0.55**	0.41*	208	29~208
**June**	0.11	0.44*	137	22~137
**July**	0.01	0.08	131	15~131
**August**	0.55**	0.61**	150	17~150
**September**	-0.07	0.26	128	15~128
**October**	0.65**	0.64**	163	18~163
**November**	0.62*	0.67**	151	16~156
**December**	0.44**	0.44*	137	22~137

The worst quality index value and the Pearson correlation coefficient between AQI and individual microblogs by month in 2012 in Beijing.

*Pearson correlation coefficient is significant at p < 0.05

**Pearson correlation coefficient is significant at p < 0.01

In addition, an interesting phenomenon was that the correlation coefficient value was usually related to the air pollution for each month, Table. 2. Higher correlation coefficient values were linked to the months with the highest worst AQI and largest range of AQI, such as r_i_ in January and April; while, the lower values were linked to the months with the lowest worst AQI and narrowest range of AQI, such as r_i_ in July and September.

#### Improvement of consistency between trends

In order to reflect the dynamics of the air quality using the individual messages for the months with low values of r_i_, we first investigated the reasons for the low values and then explored how to improve the consistency between AQI and individual messages trends when low values of r_i_ occured in the data.

**Fig 3:**
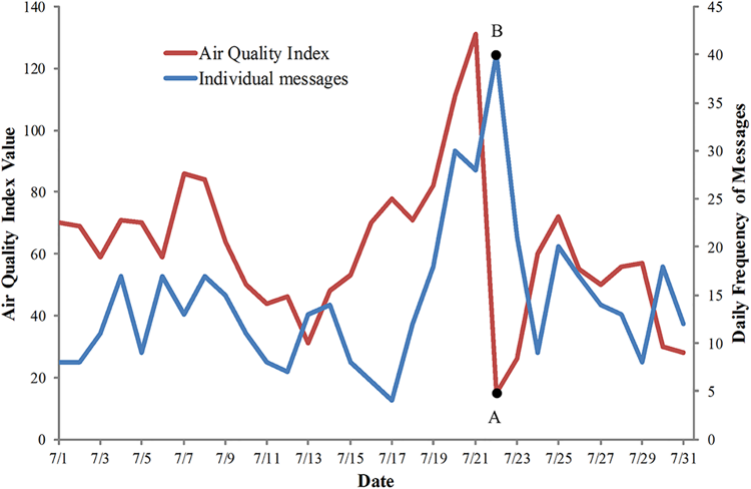
An example of the low correlation between AQI and individual messages. The trends of AQI (red line) and the individual messages (blue line) for July 2012 in Beijing. Point A is the lowest AQI value and point B is the highest daily frequency of individual messages in July.

By analyzing the sentiments expressed in individual messages in both high value months and low value months, the reasons for the low values can be ascertained qualitatively. In general, most individual messages relevant to air pollution are negative, such as“空气污染太严重了，我也要戴口罩了。”(The air pollution is too serious. I need to have a mask.). However in July with lowest value of r_i_ (0.08), many individual messages were positive, reflecting the positive attitudes about air quality. For example, “雨后，空气污染得终于没前几天那么恐怖了。”(After the rain, the air pollution is no longer a terrible problem.). The daily trends for the total individual messages and AQI in July are shown in Fig 3. The lowest recorded AQI (point A) occurred on July 22, 2012, while at the same time, the frequency of the individual messages concerning air quality was the highest (point B) in this month. Many microblogs with positive content about air quality were posted in tandem with the drop in the AQI values; thus very low AQI (best air quality) leads to many positive individual messages likely to be related to the lower correlation coefficients for certain months.

**Fig 4:**
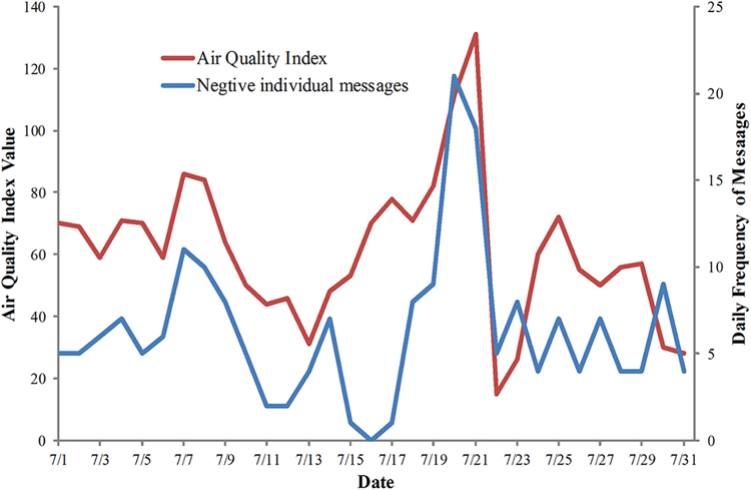
An example of the high correlation between AQI and negative individual messages. The trends of AQI (red line) and negtive individual messages (blue line) for July 2012 in Beijing.

The sentiments in individual messages were qualitatively analyzed. The microblogs were sorted into two nominal categories: positive and negative. The Pearson correlation coefficients between the AQI and the total individual messages and negative individual messages were compared. The Pearson correlation coefficient between the negative individual messages and the AQI are shown as r_ni_ in Table 3. As mentioned previously in this section, positive individual microblogs can lead to low values of r_i_. After filtering out the positive individual microblogs, the r_ni_ for each month was higher than r_i_ and all the values of r_ni_ were significant at p < 0.01. The r_ni_ obtained for the entire year was 0.62 and significant at p < 0.01. Compared to the r_i_ for the entire year, the r_ni_ was higher. Therefore, the negative individual messages were more strongly correlated to the AQI and reflect the AQI dynamics better than the individual messages. For example, the r_ni_ (0.61) for July was much higher than r_i_ (0.08) and trends of negative individual messages and AQI in this month were consistent, as shown in Fig 4.

**Table 3:** Correlation coefficient between AQI and negative individual messages.

	The Pearson correlation coefficient between AQI and the total individual messages	The Pearson correlation coefficient between AQI and the negative individual messages
**January**	0.75**	0.76**
**February**	0.65**	0.68**
**March**	0.73**	0.74**
**April**	0.83**	0.91**
**May**	0.41*	0.69**
**June**	0.44*	0.72**
**July**	0.08	0.61**
**August**	0.61**	0.66**
**September**	0.26	0.69**
**October**	0.64**	0.63**
**November**	0.67**	0.69**
**December**	0.44*	0.47**

The Pearson correlation coefficient between AQI and negative individual messages by month in 2012 in Beijing.

*Pearson correlation coefficient is significant at p < 0.05

**Pearson correlation coefficient is significant at p < 0.01

### Comparing mobile app messages to AQI

Analyzing the trends in different categories of app messages can be useful for understanding dynamics of AQI. In this study, the air quality in broadcasts produced by apps was separated into six ordinal levels: “优” (excellent), “良” (good), “轻度污染” (light pollution), “中度污染” (moderate pollution), “重度污染” (high pollution), and “严重污染” (serious pollution). We termed the app messages containing air quality levels of ‘excellent’ or ‘good’ as good app messages and the app messages containing the remaining air quality levels as pollution app messages.

**Fig 5:**
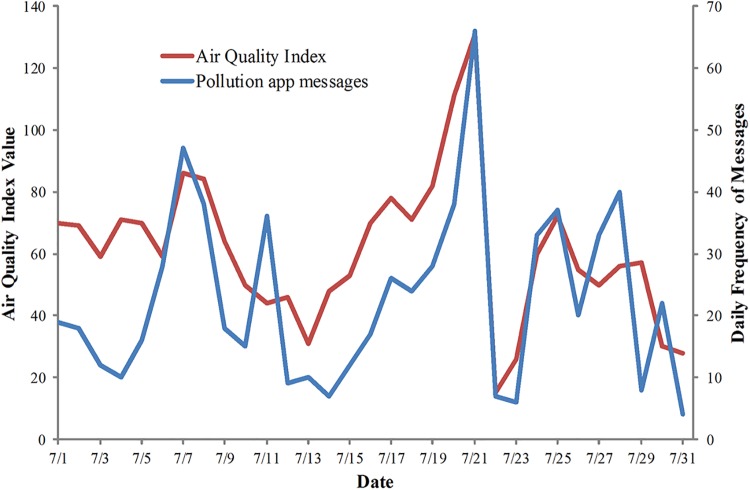
An example of high correlation between AQI and pollution app messages. The trends of AQI (red line) and pollution app messages (blue line) for July 2012 in Beijing.

The Pearson correlation coefficient between AQI and the total app messages and the pollution app messages were denoted as the r_a_ and r_p_, respectively, as shown in Table 4. Among the 12 months, the values of r_a_ in four months were higher than 0.6 and significant at p<0.01. Compared to the r_a_, the r_p_ was much larger. For example, the r_p_ (0.84) in September was higher than r_a_ (-0.30). Most of the values of r_p_ were significant at p<0.01, and the values for nine months were higher than 0.6. As the air quality deteriorated in Beijing during the year, apps announced broadcasts containing the levels of pollution. When the air quality levels reported in these broadcasts were more serious, more people reposted these broadcasts on Sina Weibo. Thus, as the AQI increased, the frequency of pollution app messages increased. So, the pollution app messages, as an indicator of public perception of air pollution, were strongly correlated to the AQI and were able to reflect the dynamics of AQI. For example, the r_p_ in July was 0.71 and the trends of pollution app messages and AQI were consistent, as shown in Fig 5.

**Table 4:** Correlation coefficient between AQI and app messages.

	The Pearson correlation coefficients between AQI and the total app messages	The Pearson correlation coefficients between AQI and the pollution app messages
**January**	0.79**	0.82**
**February**	0.14	0.28
**March**	0.48**	0.56**
**April**	0.32	0.53**
**May**	0.11	0.66**
**June**	0.35	0.79**
**July**	0.04	0.71**
**August**	0.63**	0.74**
**September**	-0.30	0.84**
**October**	0.63**	0.72**
**November**	0.63**	0.79**
**December**	0.51**	0.61**

The Pearson correlation coefficient between AQI and app messages by month in 2012 in Beijing.

**Pearson correlation coefficient is significant at p < 0.01

### Comparing retweets to AQI

Retweet microblogs are often prompted by celebrity microblogs. In this section, the Pearson correlation coefficient between the retweet microblogs and the air quality, r_w_ was calculated and potential reasons for the low values of r_w_ are discussed.

**Fig 6:**
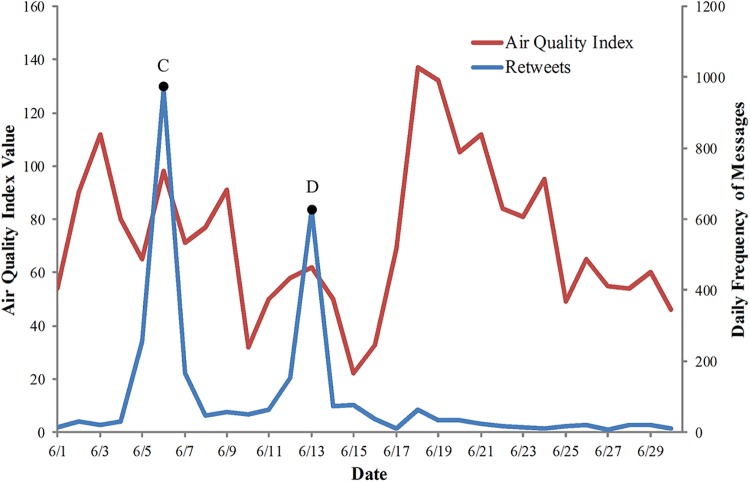
An example of the low correlation between AQI and retweets. The trends of AQI (red line) and retweet (blue line) for June 2012 in Beijing. Point C and point D are the highest two daily frequencies of retweets in June.

The r_t_ and r_w_ for each month in 2012 are shown in Table 5, and compared to the r_t_, most values of r_w_ were lower and not significant. The r_w_ for each month was relatively low indicating that the retweets were not strongly related to the AQI. Retweets were strongly correlated to the celebrity microblogs. Celebrity microblogs however, were posted sporadically and were likely unrelated to the dynamics of air quality in the real world. For example, the trends of retweets and AQI in June are shown in Fig 6. The highest two daily frequencies of retweets were on June 6 (point C) and June 13 (point D), while the AQI values for these two days were not the highest two values. By investigating the contents of retweets, the highest two frequencies of retweets were likely led by two celebrity microblogs posted over these two days.

**Table 5:** Correlation coefficient between AQI and retweets.

	The Pearson correlation coefficient between AQI and the total microblogs	The Pearson correlation coefficient between AQI and the retweets
**January**	0.70**	0.16
**February**	0.29	0.18
**March**	0.27	0.17
**April**	0.23	0.03
**May**	0.55**	0.57**
**June**	0.11	0.07
**July**	0.01	-0.04
**August**	0.55**	0.14
**September**	-0.07	0.27
**October**	0.65**	0.47**
**November**	0.62*	0.46**
**December**	0.44**	0.13

The Pearson correlation coefficient between AQI and retweets by month in 2012 in Beijing.

*Pearson correlation coefficient is significant at p < 0.05

**Pearson correlation coefficient is significant at p < 0.01

## Using Individual Messages to Monitor AQI

Based on the correlation analysis, filtered social media messages were found to be strongly correlated to the dynamics of AQI. In this section, we applied the Gradient Tree Boosting (GTB) to monitor AQI in 2013 by using individual messages. Although the app messages were able to reflect the dynamics of AQI, app messages already had AQI information and did not have monitoring value. Therefore, the app messages were not used to monitor AQI. We inputted daily frequencies of negative individual messages and positive individual messages into GTB as two explanatory variables. The data in 2012 was treated as training set to produce a monitor model. The individual messages for 2013 were sorted as positive and negative qualitatively and the data in 2013 were taken as test set.

As three important parameters in GTB, the learning rate *v*, the number of iterations ***M***, and the depth of the decision tree *S* must be decided and optimized. In this study, we optimized these parameters in two steps. First, empirically, the range of *v*, *M*, and *S* were separately set as {0.005,0.01,0.05}, {200,300,400,…,700}, {2,3,4,…12}. Second, we applied 5-fold cross-validation with data in 2012 to evaluate each set of parameters. By comparing different sets of parameters, the best values were *v* = 0.01, *M* = 400 and *S* = 7.

**Fig 7:**
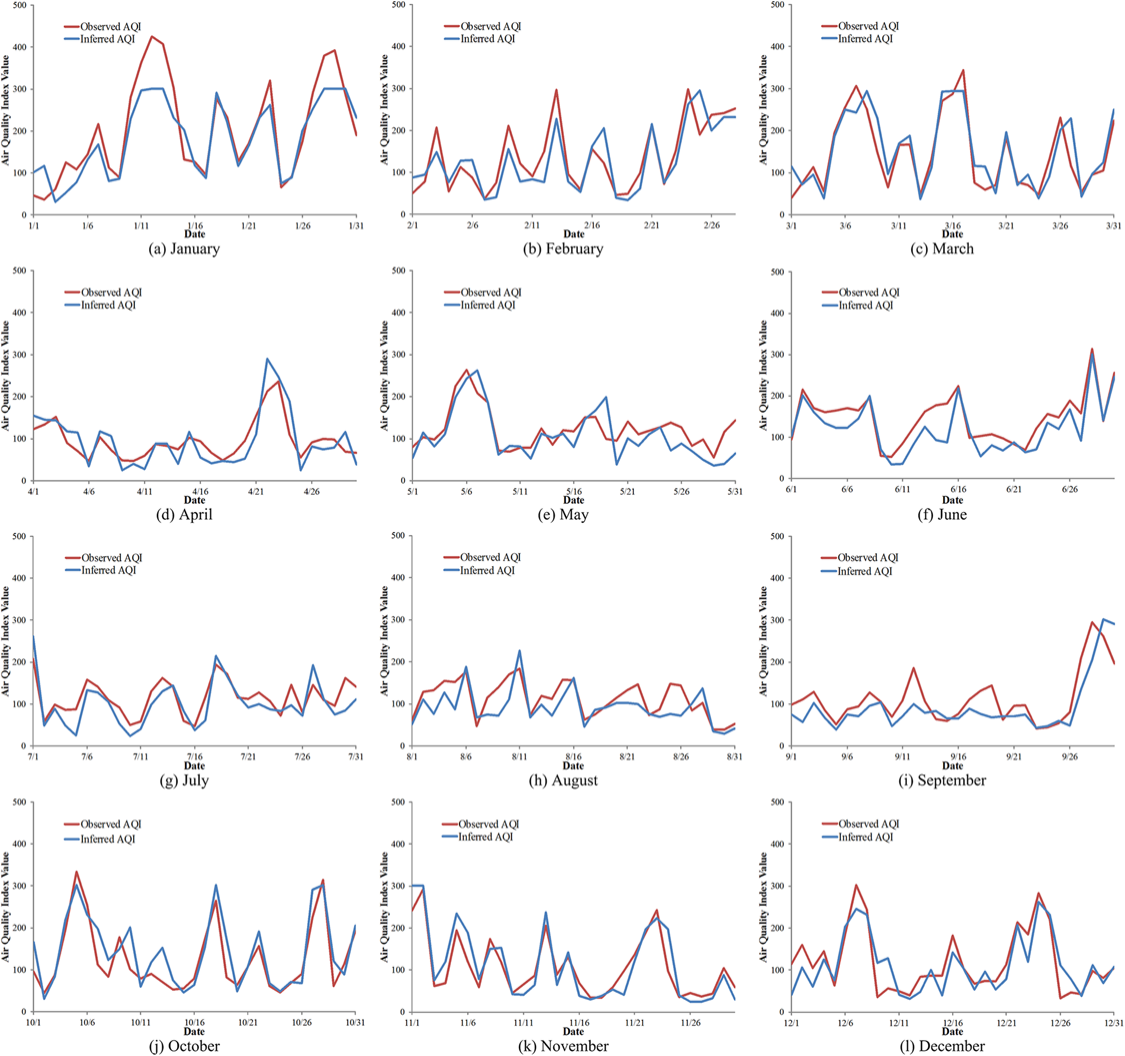
The inferred AQI and the observed AQI in each month in 2013. The trends of observed AQI (red line) and inferred AQI (blue line) in each month in 2013.

We show the RMSE for each month in 2013 in Table 6. The R^2^ of the model was 0.59. The inferred AQI did not vary more than 30.0 (on average). This error is lower than found in currently published related work. The inferred AQI and observed AQI in each month are shown in Fig 7. The trends were consistent and most values of inferred AQI were close to the observed AQI. This indicates that the social media messages can be used as a new way to monitor the dynamics of AQI. There were certain disparities between inferred AQI and observed AQI on some days, such as on January 12 and January 13, 2013. The values of AQI on these days were two top values in 2013. Analyzing data used in this study, we found that the training set lacked samples with high AQI. This means that the errors for high AQI were larger than the errors for low AQI.

**Table 6:** RMSE for each month in 2013.

	RMSE
**January**	53.0
**February**	41.5
**March**	38.6
**April**	32.8
**May**	37.9
**June**	36.8
**July**	32.0
**August**	36.0
**September**	42.0
**October**	42.8
**November**	33.9
**December**	38.6

RMSE for each month in 2013 in Beijing.

## Conclusions

Advancements of smart phone technology, mobile computing, and social media services provide opportunities for researchers to explore the inter-correlations between real-space and cyberspace. In this research, we examined such dynamic relationships by using a popular Chinese Social Media Service and focused on monitoring the air pollution in the real world. Our case study of Beijing demonstrates that the filtered messages are strongly correlated to AQI and can be used to monitor the dynamics of air pollution to some extent. In addition, the social media messages can also be considered as a method to collect information about public perceptions of air pollution. From the contents of messages, we found that people cared about preventing the deterioration of air quality, protective gear, and health risks associated with air pollution.

**Fig 8:**
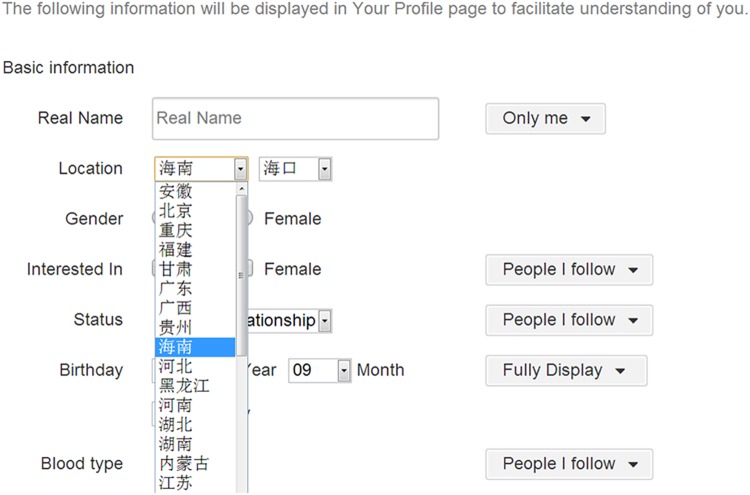
The page of Sina Weibo users’ profile. Basic information of user’s profile. The user-defined geolocation of Sina Weibo users is selected from the drop-down list and different from the Twitter with open entry fields.

This study illustrates that multi-step filtering and classifying procedures for social media messages are the key components when building a successful monitoring and tracking model for air pollution. Although our preliminary analysis suggests a promising method for monitoring of air quality in major cities, we need to be very careful in developing practical applications of social media analytics in the future. There are a few challenges and potential problems in our future applications:

1. Improvement of the model. On the days with high AQI, certain disparities between inferred AQI and observed AQI were identified. In the future work, we will collect more samples with high AQI and improve the ability of our model to infer high values.
2. Changes in popular social media platforms in China. When we started this research in 2012, Weibo was the most popular social media platform in China. Subsequently, a new platform, “微信” (WeChat), by 2014, had become the most popular social media and real-time messenger platform. However, “微信” (WeChat) does not provide researcher-friendly APIs for collecting user messages directly by keywords or users locations.
3. Locational uncertainty in user profiles. When we collected the messages within the city of Beijing, we used user registration profiles. Sina Weibo provides a very detailed location dropdown-list for users to select which province or city and which sub-region they are located in, as shown in Fig 8. However, there may be some errors and uncertainty in the real locations of users. Further studies are needed to estimate the probability and uncertainty of locational information in user profiles.
4. Automatic procedures for analysis of sentiments expressed in messages. Our study used a manual qualitative classification method to separate positive and negative sentiments in the original individual messages. Although this process can improve the correlation results, it is very time consuming and labor intensive. We need to develop machine learning procedures to conduct analysis of sentiments.
5. Privacy issues. Some social media messages are very personal and may reveal sensitive personal information. Many social media users are not aware that their messages are publicly searchable. They may be surprised to see their messages used in published research. Currently, academic ethical standards and public information laws are not well-defined in the domain of social media research. Our research community needs to have more discussions to provide better guidance about the legal and privacy issues associated with social media research and applications.

One interesting finding in this research was that the worst month of air quality (April, 2012) generated the highest correlation coefficient between the filtered social media messages and AQI. One possible explanation adopts the Shannon-Hartley information theorem [35]. The Shannon-Hartley information theorem defines the channel capacity (C) via a communication channel (the bandwidth of a medium: B) with the impacts of noise interference (N) and signal power (S) during the communication process (from a transmitter to a receiver). When S (signal power) becomes stronger, the channel capacity is bigger. When N (noise) is bigger, the channel capacity becomes smaller. We could apply the Shannon-Hartley information theorem in this study. In April 2012, the signals of air pollutions from social media users (S) are the strongest. Therefore, the channel capacity (C) transferred via the Sina Weibo (medium: B) is the highest. The temporal changes of daily air quality can be transferred effectively (with less noise) via the social media channel (Weibo). Therefore, the correlation efficient becomes the highest comparing to other months (with weaker signals). Although this assumption is just one of the possible explanations of our findings, future studies are required to verify our hypothesis.
